# The effect of governance structures on optimal control of two-patch epidemic models

**DOI:** 10.1007/s00285-023-02001-8

**Published:** 2023-10-20

**Authors:** Emily Howerton, Kyle Dahlin, Christina J. Edholm, Lindsey Fox, Margaret Reynolds, Brandon Hollingsworth, George Lytle, Melody Walker, Julie Blackwood, Suzanne Lenhart

**Affiliations:** 1 Department of Biology and Center for Infectious Disease Dynamics, Pennsylvania State University, University Park, PA USA; 2 Center for the Ecology of Infectious Diseases, Odum School of Ecology, University of Georgia, Athens, GA USA; 3 Mathematics Department, Scripps College, Claremont, CA USA; 4 Mathematics Discipline, Eckerd College, Saint Petersburg, FL, USA; 5 Department of Mathematical Sciences, United States Military Academy, West Point, NY, USA; 6 Department of Entomology, Cornell University, Ithaca, NY, USA; 7 Department of Biology, Chemistry, Mathematics, and Computer Science, University of Montevallo, Montevallo, AL USA; 8 Department of Medicine, University of Florida, Gainesville, FL USA; 9 Department of Mathematics and Statistics, Williams College, Williamstown, MA USA; 10 Department of Mathematics, University of Tennessee, Knoxville, TN USA

**Keywords:** Governance, ODE model, Optimal control, Spatial movement, Cholera, Ebola, 92-10

## Abstract

Infectious diseases continue to pose a significant threat to the health of humans globally. While the spread of pathogens transcends geographical boundaries, the management of infectious diseases typically occurs within distinct spatial units, determined by geopolitical boundaries. The allocation of management resources within and across regions (the “governance structure”) can affect epidemiological outcomes considerably, and policy-makers are often confronted with a choice between applying control measures uniformly or differentially across regions. Here, we investigate the extent to which uniform and non-uniform governance structures affect the costs of an infectious disease outbreak in two-patch systems using an optimal control framework. A uniform policy implements control measures with the same time varying rate functions across both patches, while these measures are allowed to differ between the patches in a non-uniform policy. We compare results from two systems of differential equations representing transmission of cholera and Ebola, respectively, to understand the interplay between transmission mode, governance structure and the optimal control of outbreaks. In our case studies, the governance structure has a meaningful impact on the allocation of resources and burden of cases, although the difference in total costs is minimal. Understanding how governance structure affects both the optimal control functions and epidemiological outcomes is crucial for the effective management of infectious diseases going forward.

**Supplementary Information:**

The online version contains supplementary material available at 10.1007/s00285-023-02001-8.

## Introduction

Infectious disease management remains a critical challenge as both existing and emerging infectious diseases increasingly burden communities worldwide (Daszak et al. 2000; Jones et al. 2008; Morens and Fauci 2012; Global burden 2019). Recent advances in pharmaceutical and non-pharmaceutical interventions have improved the ability of communities to respond to outbreaks (Morse et al. 2012; Flaxman et al. 2020; Lal et al. 2022). However, the implementation of successful infectious disease management policies is constrained by political boundaries, while infectious agents often freely spread across the same borders (e.g. due to immigration, the movement of livestock, the flow of water, or the dispersal of insect vectors) (Mirkovic et al. 2014; Bwire et al. 2016; Hemida et al. 2017; Agusto et al. 2021). Consequently, a key consideration for successful disease management should be the allocation of resources within and across these discrete spatial units.

Mathematical models are often used by researchers and public health officials to compare the effectiveness of various disease management policies (Martcheva 2013; Feng 2014; Brauer et al. 2019). Typically based on the classic Susceptible–Infected–Recovered or *SIR* model paradigm (Kermack and McKendrick 1927; Anderson and May 1991), these transmission models can be coupled with optimal control theory to determine the most efficient implementation of a management strategy accounting for cost (e.g. the cost of infections or the cost of response) (Lenhart and Workman 2007). Many models consider space implicitly, so that both the disease dynamics and associated management policies are homogeneous across space (Miller Neilan et al. 2010; Bonyah et al. 2016)). However in cases of cross-boundary pathogen transmission, evaluating the effectiveness of disease management policies requires that models explicitly include space. Here, we use the term *patches* to generically refer to neighboring jurisdictions, which can take the form of states, countries, or any other political or cultural subdivisions. We assume that these patches are connected in some way, for example by the movement of individuals, and need not be adjacent in a geographic sense. In considering such a structure, many natural questions arise. For example, is it better for two patches to implement the same or different management policies? How does this decision depend on differences between the properties of each patch?

In deterministic optimal control models, spatial features may be represented in continuous space using partial differential equations (Ding et al. 2012; Fitzgibbon et al. 2020) or in discrete space using ordinary differential and discrete equations (Ding et al. 2007). For example, many studies determine optimal resource allocation for a particular type of control strategy, such as vaccination (Asano et al. 2008; Miyaoka et al. 2019). Such studies typically aim to minimize the overall total costs across all patches, including the costs of new cases, the number of current infected individuals, and/or control implementation (Miller Neilan et al. 2010; Kelly et al. 2016; Lee et al. 2020b). These policies may be non-uniform, meaning that the optimal control applied to one patch need not be the same as in the others. However, this approach does not consider constraints that may be faced by decision makers managing multiple jurisdictions. For example, a decision maker responsible for multiple patches may choose to apply uniform policies across patches motivated by fairness or equity. Consequently, it is possible that such an “equitable”, uniform policy may not optimally minimize overall total cost.

Recently, Blackwood et al. (2021) began to tackle the question of how governance structure impacts optimal control as a Structured Decision Making problem (Gregory et al. 2012; Shea et al. 2014). This work considered a generic disease that is highly transmissible, modeled within the *SIR*-model paradigm, and evaluated the trade-offs between local and global decision-making for multiple management strategies, including time-constant implementation of vaccination, medication and travel restrictions. The work of Blackwood et al. (2021) highlighted the potential trade-offs between the local and global management of a generic infectious disease, showing that policies which are best for one jurisdiction may lead to worse disease outcomes overall. We extend this work in three primary ways: (1) we consider the management of two specific pathogens with differing modes of transmission; (2) we optimize management strategies with time-varying functions using optimal control theory (with disease outcomes combined with cost of implementing the controls), and (3) we compare the optimal control decisions under both uniform and non-uniform governance structures following a method from Sanchirico et al. (2021), a fisheries management study. We define a uniform policy as the implementation of exactly the same level of control in both patches (“centralized uniform management” in Blackwood et al. 2021), and a non-uniform policy as one in which the level of control for each patch is allowed to differ (“centralized jurisdiction-specific management” in Blackwood et al. (2021)).

To compare the effect of multiple governance structures on pathogens with different modes of transmission, we develop two-patch *SIR*-type models for cholera and Ebola. Cholera is transmitted primarily through water, its environmental reservoir, though it can also be directly transmitted (Fitzgibbon et al. 2020; Legros 2018). In contrast, Ebola is transmitted directly from both infected individuals and from direct contact with the corpses of individuals that died from Ebola and have not had a sanitary burial (Dowell et al. 1999). We focus on these disease systems because cross-boundary transmission has occurred and is considered an important consideration for the prevention and control of outbreaks (Mirkovic et al. 2014; Bwire et al. 2016). Furthermore, these diseases have well-established models that reliably capture their transmission dynamics, allowing us to focus on optimal control of each disease rather than model validation (Tien and Earn 2010; Kelly et al. 2016; Burton et al. 2021). Investigating these types of governance management features is a novel application of optimal control theory. For each disease example, we model the control of transmission through two distinct and concurrent management interventions, specifically vaccination and sanitation for cholera and vaccination and hospitalization for Ebola. The Pontryagin Maximum Principle (Pontryagin et al. 1962) is used to characterize the optimal controls and their corresponding adjoint functions. We then consider the numerical results obtained from simulations of both models. Finally, we compare the results of the two models to evaluate important differences in how the choice of governance structure impacts the optimal control of each disease.

## Models

### Cholera

Our cholera model represents two geographically distinct populations, called patches, each divided into the epidemiological sub-compartments of susceptible, *S*, infectious, *I*, and recovered, *R*, with *N* representing the total population size, $$ N = S + I + R$$. Since cholera is primarily spread through contact with contaminated water (Fitzgibbon et al. 2020; Legros 2018), we include a compartment, *W*, representing cholera bacteria in the water supply. The number of newly infected individuals depends on both the number of currently infected individuals and the amount of cholera bacteria in the water.

**Fig. 1:**
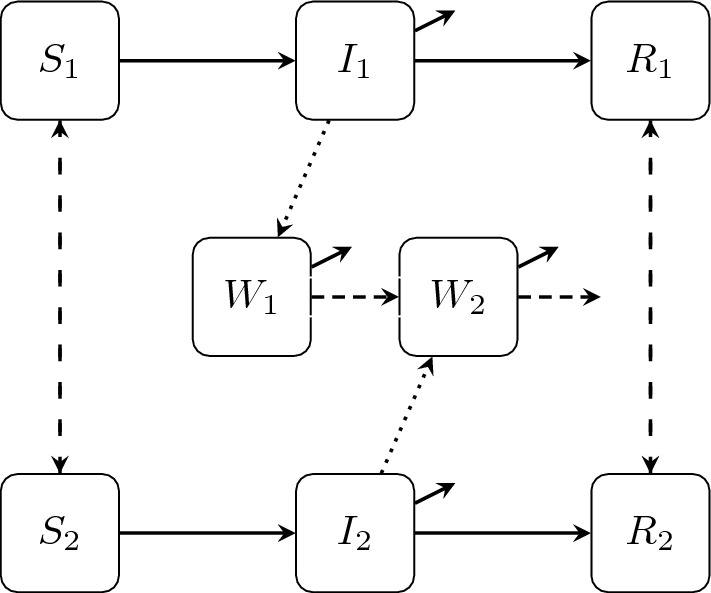
Schematic of cholera transmission within and between Patch 1 and Patch 2. Susceptible individuals, $S_i$, become infected based on the number of infected individuals, $I_i$, and the amount of cholera-contaminated water, $W_i$. Infected individuals either recover, $R_i$, or experience disease-induced mortality. Susceptible and recovered individuals migrate between patches while infected individuals remain stationary due to the severity of their symptoms. Contaminated water in Patch 1, $W_1$, decays or flows into Patch 2, $W_2$, where contaminated water either decays or flows out of the system. In the diagram, solid lines represent epidemiological transitions, dotted lines represent shedding, and dashed lines represent movement

Non-infected individuals (i.e., susceptible and recovered) can move between the two patches; however, because the symptoms of cholera make travel difficult (Melbourne 2011), we assume there is no movement of infected individuals between patches. We model the migration of individuals between patches with an Eulerian modeling approach (Cosner 2015; Vargas Bernal et al. 2022). We assume that contaminated water is transferred by a water source connecting the patches (e.g., a river or stream), where the second patch is downstream from the first patch (i.e., water flows from Patch 1 to Patch 2). Our model is based on the models introduced in Kelly et al. (2016) and Tien and Earn (2010). For comparisons of several cholera models with vaccination scenarios, see the work of Lee et al. (2020a). A schematic of the model is given in Fig. 1, and the system of ordinary differential equations describing transmission and control dynamics is given by (1).

1 $$\begin{aligned} \begin{aligned} S_1'&= \mu _1N_1 - \beta _{I1}S_1I_1 - (1-u_1(t))\beta _{W1}S_1W_1 - \mu _1S_1 - v_1(t)S_1 - m_1S_1 + m_2S_2 \\ I_1'&= \beta _{I1}S_1I_1 + (1-u_1(t))\beta _{W1}S_1W_1 - (\mu _1+\gamma _1+\delta _1)I_1 \\ R_1'&= \gamma _1I_1 - \mu _1R_1 + v_1(t)S_1 - m_1R_1 + m_2R_2 \\ W_1'&= \xi _1I_1 - \nu _1W_1 - \rho _1W_1 \\ S_2'&= \mu _2N_2 - \beta _{I2}S_2I_2 - (1-u_2(t))\beta _{W2}S_2W_2 - \mu _2S_2 - v_2(t)S_2 + m_1S_1 - m_2S_2 \\ I_2'&= \beta _{I2}S_2I_2 + (1-u_2(t))\beta _{W2}S_2W_2 - (\mu _2+\gamma _2+\delta _2)I_2 \\ R_2'&= \gamma _2I_2 - \mu _2R_2 + v_2(t)S_2 + m_1R_1 - m_2R_2 \\ W_2'&= \xi _2I_2 - \nu _2W_2 + \rho _1W_1 - \rho _2W_2 \end{aligned} \nonumber \\ \end{aligned}$$

We assume that cholera transmission can be controlled through two means: the sanitation of contaminated water and the vaccination of susceptible individuals. Reducing transmission from contaminated water has been achieved by the provision of alternative sources of clean water (Legros 2018) or sanitation tablets or other technology which sanitize water at the point of contact (Sévère et al. 2016). Therefore, we model our water sanitation control, represented by $u_i(t)$, as a percent reduction in the rate of transmission from the water compartments (i.e., $0 \le u_i(t) \le 1$). In each patch, we also introduce vaccination as another form of transmission control (Sévère et al. 2016). The vaccination controls, $v_i(t)$, represent a per-capita rate of vaccination, which permanently immunizes susceptible individuals and prevents future infection in those individuals. With these two controls, we seek to find the optimal control vector that minimizes the objective functional given in (2).

2 $$\begin{aligned}{} & {} J(u_1, v_1, u_2, v_2) \nonumber \\{} & {} \quad = J_1(u_1,v_1) + J_2(u_2,v_2) \nonumber \\{} & {} \quad = \int _0^T \Big [ b_1(\beta _{I1}S_1I_1 + (1-u_1)\beta _{W1}S_1W_1) + A_1v_1S_1 + \epsilon _1v^2_1 + B_1u_1 + \eta _1u_1^2 \Big ] dt \nonumber \\{} & {} \qquad + \int _0^T \Big [ b_2(\beta _{I2}S_2I_2 + (1-u_2)\beta _{W2}S_2W_2) + A_2v_2S_2 + \epsilon _2v^2_2 + B_2u_2 + \eta _2u_2^2 \Big ] dt\nonumber \\ \end{aligned}$$

The objective functional (2) represents the cost of the total number of new cases and the cost of implementing both controls, including nonlinear, quadratic costs. Since the *relative* sizes of the coefficients in *J* determine the optimal control, the cost coefficients of new cases, $b_1$ and $b_2$, are set to one. The costs of sanitation are represented by the weights $B_1$, $B_2$, $\eta _1$ and $\eta _2$ and the costs of vaccination are represented by the weights $A_1$, $A_2$, $\epsilon _1$ and $\epsilon _2$.

The control set for the *non-uniform policy*, in which each patch can respond to an outbreak using different controls, is given by (3).

3 $$\begin{aligned} U= & {} \{ (u_1, v_1, u_2, v_2) \in [ L^\infty (0, T)]^4 \,\, | \,\, 0 \le u_i(t)\nonumber \\\le & {} u_{i,\max }, \,\, 0 \le v_i(t) \le v_{i,\max }, i=1,2 \} \end{aligned}$$

An optimal control vector, $(u_1^*, v_1^*, u_2^*, v_2^*)$, will minimize the total cost of cases and controls, and satisfy (4).

4 $$\begin{aligned} J(u_1^*, v_1^*, u_2^*, v_2^*) = \min _U J(u_1, v_1, u_2, v_2) \end{aligned}$$

Because the controls, state variables and the derivatives of the state variables are all bounded, standard compactness results imply the existence of an optimal control vector, $(u_1^*, v_1^*, u_2^*, v_2^*)$, for this problem (Fleming and Rishel 1975; Kelly et al. 2016). With this existence result, we use Pontryagin’s Maximum Principle to obtain the optimal control characterization shown in (5).

5 $$\begin{aligned} \begin{aligned} u_1^*(t)&= \min \Bigg \{ u_{1,\max }, \,\, \max \Big \{ 0, \,\, \frac{b_1\beta _{W1}S_1W_1 - B_1 - \lambda _1\beta _{W1}S_1W_1 + \lambda _2\beta _{W1}S_1W_1}{2\eta _1} \Big \} \Bigg \} \\ u_2^*(t)&= \min \Bigg \{ u_{2,\max }, \,\, \max \Big \{ 0, \,\, \frac{b_2\beta _{W2}S_2W_2 - B_2 - \lambda _5\beta _{W2}S_2W_2 + \lambda _6\beta _{W2}S_2W_2}{2\eta _2} \Big \} \Bigg \} \\ v_1^*(t)&= \min \Bigg \{ v_{1,\max }, \,\, \max \Big \{ 0, \,\, \frac{\lambda _1S_1 - A_1S_1 - \lambda _3S_1}{2\epsilon _1} \Big \} \Bigg \} \\ v_2^*(t)&= \min \Bigg \{ v_{2,\max }, \,\, \max \Big \{ 0, \,\, \frac{\lambda _5S_2 - A_2S_2 - \lambda _7S_2}{2\epsilon _2} \Big \} \Bigg \}, \end{aligned}\nonumber \\ \end{aligned}$$

The adjoint functions, $\lambda _i$ for $i=1,...,8$, their derivatives and final time conditions, and the Hamiltonian obtained from Pontryagin’s Maximum Principle used to obtain the optimal control characterization are discussed and given in full in “Appendix B.1”

If we instead insist on a policy wherein the same level of controls is used in both patches, which we deem a *uniform policy*, the control set is then given by (6), where $u_1 = u_2 = u$ and $v_1 = v_2 = v$.

6 $$\begin{aligned} U = \{ (u, v) \in [ L^\infty (0, T)]^2 \,\, | \,\, 0 \le u(t) \le u_{\max }, \,\, 0 \le v(t) \le v_{\max } \} \end{aligned}$$

In this case, the characterization of an optimal control vector $(u^*, v^*)$ is given by (7).

7 $$\begin{aligned} u^*= & {} \min \Bigg \{ u_{\max }, \, \max \Big \{ 0, \, {{\frac{\begin{array}{c}b_1\beta _{W1}S_1W_1 - B_1 - \lambda _1\beta _{W1}S_1W_1 + \lambda _2\beta _{W1}S_1W_1\\ + b_2\beta _{W2}S_2W_2 - B_2 - \lambda _5\beta _{W2}S_2W_2 + \lambda _6\beta _{W2}S_2W_2\end{array}}{2(\eta _1 + \eta _2)} }} \Big \} \Bigg \} \nonumber \\ v^*= & {} \min \Bigg \{ v_{\max }, \,\, \max \Big \{ 0, \,\, \frac{\lambda _1S_1 - A_1S_1 - \lambda _3S_1 + \lambda _5S_2 - A_2S_2 - \lambda _7S_2}{2(\epsilon _1 + \epsilon _2)} \Big \} \Bigg \} \end{aligned}$$

See Sect. 3.1 for details on the implementation of this model numerically, including parameterization (Table 1).

### Ebola virus disease

Similar to our cholera model, we include transmission within and between two patches in our Ebola model. Each population is divided into the epidemiological sub-compartments: susceptible, *S*, exposed, *E*, infectious, *I*, hospitalized, *H*, and recovered, *R*, with *N* representing the total population size, $$ N = S + E + I + H + R$$. A significant transmission pathway of Ebola is direct contact with the corpse of someone who has recently died of the disease (Dowell et al. 1999). We therefore also include the compartment *D* to represent the amount of infectious corpses in the system which have not received a sanitary burial. We assume that hospitalized individuals are medically isolated and thus do not contribute to transmission. Furthermore, if an individual infected with Ebola dies while hospitalized, we assume that they are buried in a sanitary manner and thus do not contribute to the *D* compartment. Our model is based on the model introduced in Burton et al. (2021).

**Fig. 2:**
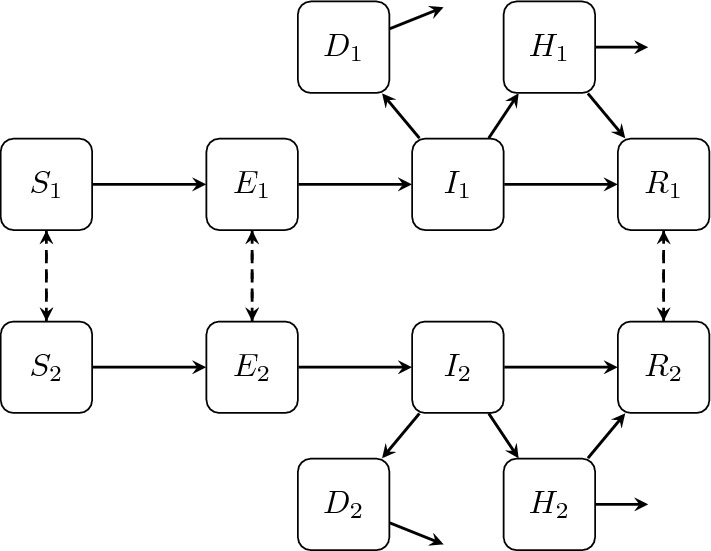
Schematic of Ebola transmission within and between Patch 1 and Patch 2. Susceptible individuals, $S_i$, become exposed, $E_i$, and eventually infectious, $I_i$. Infected individuals may recover, $R_i$, become hospitalized, $H_i$, or, if not hospitalized, die from the disease and potentially contribute to onward transmission, $D_i$. Susceptible, exposed and recovered individuals may migrate between patches. However, infectious individuals do not travel due to the severity of their symptoms. Hospitalized individuals are assumed to be medically isolated, hence they do not travel between patches and do not contribute to onward transmission. Both infectious and hospitalized individuals experience disease induced mortality, with a reduced mortality rate for hospitalized individuals. Solid lines represent epidemiological transitions, and dashed lines represent movement

We assume non-infected individuals (i.e., susceptible, exposed and recovered) move between patches; however, because of the severity of disease associated with Ebola virus infection (Weyer et al. 2015), we assume infected individuals do not travel between patches. We model the migration of individuals between patches with an Eulerian modeling approach (Cosner 2015; Vargas Bernal et al. 2022). A schematic of the model is given in Fig. 2 and the system of ordinary differential equations describing transmission and control dynamics is given by (8).

8 $$\begin{aligned} S_1'= & {} \mu _1N_1 - \beta _{I1}S_1I_1 - \beta _{D1}S_1D_1 - \mu _1 S_1 - v_1(t)S_1 - m_1S_1 + m_2S_2 \nonumber \\ E_1'= & {} \beta _{I1}S_1I_1 + \beta _{D1}S_1D_1 - (\mu _1 + \alpha _1)E_1 - m_1E_1 + m_2E_2 \nonumber \\ I_1'= & {} \alpha _1E_1 - (\mu _1 + \gamma _{I1} + (1+u_1(t))\varphi _1 + \delta _{I1} )I_1 \nonumber \\ H_1'= & {} (1 + u_1(t))\varphi _1I_1 - (\mu _1 + \gamma _{H1} + \delta _{H1})H_1 \nonumber \\ D_1'= & {} \delta _{I1}I_1 - \xi _1D_1 \nonumber \\ R_1'= & {} v_1(t)S_1 + \gamma _{I1}I_1 + \gamma _{H1}H_1 - \mu _1R_1 - m_1R_1 + m_2R_2 \nonumber \\ S_2'= & {} \mu _2N_2 - \beta _{I2}S_2I_2 - \beta _{D2}S_2D_2 - \mu _2 S_2 - v_2(t)S_2 + m_1S_1 - m_2S_2 \nonumber \\ E_2'= & {} \beta _{I2}S_2I_2 + \beta _{D2}S_2D_2 - (\mu _2 + \alpha _2)E_2 + m_1E_1 - m_2E_2 \nonumber \\ I_2'= & {} \alpha _2E_2 - (\mu _2 + \gamma _{I2} + (1 + u_2(t))\varphi _2 + \delta _{I2} )I_2 \nonumber \\ H_2'= & {} (1 + u_2(t))\varphi _2I_2 - (\mu _2 + \gamma _{H2} + \delta _{H2})H_2 \nonumber \\ D_2'= & {} \delta _{I2}I_2 - \xi _2D_2 \nonumber \\ R_2'= & {} v_2(t)S_2 + \gamma _{I2}I_2 + \gamma _{H2}H_2 - \mu _2R_2 + m_1R_1 - m_2R_2 \end{aligned}$$

In our model, the transmission of Ebola is controlled through the hospitalization of infected individuals and the vaccination of susceptible individuals. Tactics to slow the spread of Ebola have centered around increasing the number of infected individuals that are cared for in well-equipped facilities (e.g., increased personal protective wear and training for healthcare workers, or increasing the number of hospital beds at regional Ebola treatment centers (Bell et al. 2016; WHO 2014)). Because the hospitalized compartment, *H*, represents individuals in such facilities, we model these control efforts, $u_i(t)$, as a proportional increase in the per-capita hospitalization rate of infectious individuals. In addition, vaccination, represented by per-capita rates $v_i(t)$, permanently immunizes susceptible individuals. Using these two controls, we seek to find the optimal control vector that minimizes the objective functional given by (9).

9 $$\begin{aligned}{} & {} J(u_1, v_1, u_2, v_2) \nonumber \\{} & {} \quad = J_1(u_1,v_1) + J_2(u_2,v_2) \nonumber \\{} & {} \quad = \int _0^T \Big [ b_1(\beta _{I1}S_1I_1 + \beta _{D1}S_1D_1) + A_{1}v_1(S_1 + E_1) + \epsilon _{1}v_1^2 + B_{1}u_1\varphi _1I_1 + \eta _{1}u_1^2 \Big ] dt\nonumber \\{} & {} \qquad + \int _0^T \Big [ b_2(\beta _{I2}S_2I_2 + \beta _{D2}S_2D_2) + A_{2}v_2(S_2 + E_2) + \epsilon _{2}v_2^2 + B_{2}u_2\varphi _2I_2 + \eta _{2}u_2^2 \Big ] dt \nonumber \\ \end{aligned}$$

The objective functional (9) represents the cost of the total number of new cases together with the cost of implementing the controls, including nonlinear, quadratic costs. The costs of new cases are represented by the weights $b_1$ and $b_2$, and are set to one. The costs of hospitalization are represented by $B_1$, $B_2$, $\eta _1$ and $\eta _2$, while the costs of vaccination are represented by the weights $A_1$, $A_2$, $\epsilon _1$ and $\epsilon _2$. The values of the control cost coefficients are given in Table 3.

The control set for the *non-uniform policy* is defined in (10).

10 $$\begin{aligned} U= & {} \{ (u_1, v_1, u_2, v_2) \in [ L^\infty (0, T)]^4 \,\, | \,\, 0 \le u_i(t)\nonumber \\ {}\le & {} u_{i,\max }, \,\, 0 \le v_i(t) \le v_{i,\max }, i=1,2 \} \end{aligned}$$

An optimal control vector $(u_1^*, v_1^*, u_2^*, v_2^*)$ satisfies (11).

11 $$\begin{aligned} J(u_1^*, v_1^*, u_2^*, v_2^*) = \min _U J(u_1, v_1, u_2, v_2) \end{aligned}$$

As in the cholera case, the optimal control vector exists, so we can apply Pontryagin’s Maximum Principle to form the Hamiltonian, and obtain the resulting adjoint differential equations and optimal control characterization $(u_1^*, v_1^*, u_2^*, v_2^*)$ in (12) (Pontryagin et al. 1962). The Hamiltonian and adjoint differential equations are given in full in “Appendix B.2”

12 $$\begin{aligned} \begin{aligned} u_1^*(t)&= \min \Bigg \{ u_{1,\max }, \,\, \max \Big \{ 0, \,\, \frac{-B_1\varphi _1I_1 + \lambda _3\varphi _1I_1 - \lambda _4\varphi _1I_1}{2\eta _1} \Big \} \Bigg \} \\ u_2^*(t)&= \min \Bigg \{ u_{2,\max }, \,\, \max \Big \{ 0, \,\, \frac{-B_2\varphi _2I_2 + \lambda _9\varphi _2I_2 - \lambda _{10}\varphi _2I_2}{2\eta _2} \Big \} \Bigg \} \\ v_1^*(t)&= \min \Bigg \{ v_{1,\max }, \,\, \max \Big \{ 0, \,\, \frac{-A_1(S_1 + E_1) + \lambda _1S_1 - \lambda _6S_1}{2\epsilon _1} \Big \} \Bigg \} \\ v_2^*(t)&= \min \Bigg \{ v_{2,\max }, \,\, \max \Big \{ 0, \,\, \frac{-A_2(S_2 + E_2) + \lambda _7S_2 - \lambda _{12}S_2}{2\epsilon _2} \Big \} \Bigg \} \end{aligned} \end{aligned}$$

In the case of a *uniform policy*, the control set is given by (13) and the characterization of an optimal control vector $(u^*, v^*)$ is given by (14), where $u_1 = u_2 = u$ and $v_1 = v_2 = v$.

13 $$\begin{aligned} U= & {} \{ (u, v) \in [ L^\infty (0, T)]^2 \,\, | \,\, 0 \le u(t) \le u_{\max }, \,\, 0 \le v(t) \le v_{\max } \}, \end{aligned}$$

14 $$\begin{aligned} u^*= & {} \min \Bigg \{ u_{\max }, \,\, \max \Big \{ 0, \,\, \frac{-B_1\varphi _1I_1 + \lambda _3\varphi _1I_1 - \lambda _4\varphi _1I_1 - B_2\varphi _2I_2 + \lambda _9\varphi _2I_2 - \lambda _{10}\varphi _2I_2}{2(\eta _1 + \eta _2)} \Big \} \Bigg \} \nonumber \\ v^*= & {} \min \Bigg \{ v_{\max }, \,\, \max \Big \{ 0, \,\, \frac{-A_1(S_1 + E_1) + \lambda _1S_1 - \lambda _6S_1 - A_2(S_2 + E_2) + \lambda _7S_2 - \lambda _{12}S_2}{2(\epsilon _1 + \epsilon _2)} \Big \} \Bigg \}\nonumber \\ \end{aligned}$$

## Numerical simulations

All simulations and calculations were conducted in R (R Core Team 2021). Solutions to the systems of ODEs were numerically estimated using the lsoda function in R (Soetaert et al. 2010). A forward-backward sweep algorithm was used to solve the optimality systems of the two disease models (Lenhart and Workman 2007). Other algorithms and software programs such as GPOPS and PASA have been developed to handle particular types of optimal control problems (Hager and Zhang 2016; Patterson and Rao 2014). The code used to conduct the analyses and generate the figures in this manuscript is publicly available on GitHub at https://github.com/eahowerton/governance-and-disease-control.

### Cholera

Our numerical simulations use parameters adapted from Kelly et al. (2016), which are based on data originally reported in Tuite et al. (2011) (Table 1). Because the simulation period was brief (200 days), we set the natural birth/death rate parameter, $\mu _i$, to zero. To ensure that our model approximated realistic outbreak sizes, we calculated the basic reproduction number, ${\mathcal {R}}_0$, of the two-patch system using the next-generation matrix method introduced by Diekmann et al. (2000) and explicated in van den Driessche and Watmough (2002). For the full derivation of ${\mathcal {R}}_0$ for this model, see “Appendix A.1”. With the parameters in Table 1, we have ${\mathcal {R}}_0= 2.57$, which is in line with ${\mathcal {R}}_0$ estimates from previous studies (Che et al. 2021; Mukandavire et al. 2011).

We assume each patch has a population of 100,000 individuals, an outbreak begins in Patch 1, and controls are implemented after 60 days. To implement this, we ran our model without control for an initially naive population with 100 infected individuals in Patch 1 (i.e., $S_1(0) = 99{,}900$, $S_2(0) = 100{,}000$, $I_1(0) = 100$, $I_2(0) = 0$, $R_1(0) = R_2(0) = 0$, and $W_1(0) = W_2(0) = 0$). Then, we use the size of each compartment at day 60 as initial conditions for all optimal control analyses ($S_1(60) = 77{,}528$, $S_2(60) = 93{,}546$, $I_1(60) = 4275$, $I_2(60) = 1833$, $R_1(60) = 18{,}187$, $R_2(60) = 4584$, $W_1(60) = 430$, and $W_2(60) = 176$). During this period, there were 28,925 cases and 46 deaths across both patches.

For the optimal control analyses, the linear costs for implementing the vaccination controls, $A_1$ and $A_2$, are set to 0.125, or 12.5% of the cost of cases. The upper bound for vaccination, $v_1(t)$ and $v_2(t)$, is 0.015, meaning that at most 1.5% of the population can be vaccinated per day. The costs of sanitation measures are one order of magnitude smaller than vaccination, i.e., $B_1 = B_2 = 0.0125$, or 1.25% of the cost of cases. We set the upper bound for sanitation, $u_{1}(t)$ and $u_{2}(t)$, at 0.4, meaning the transmission rate from the water reservoir can decrease by at most 40%, which is sufficiently high enough to reduce ${\mathcal {R}}_0$ below one. The full set of baseline parameters are summarized in Table 1.

**Table 1:** Symbols, descriptions and values of the baseline cholera model parameters

		Description	Value	Reference
Disease	$\mu _i$	Natural birth/death rate in Patch *i*	0 day$^{-1}$	(c)
dynamics	$\beta _{Ii}$	Transmission rate from direct contact in Patch *i*	$2.64\times 10^{-6}$ ind$.^{-1}$ day$^{-1}$	(b)
	$\beta _{Wi}$	Transmission rate from contaminated water in Patch *i*	$1.01\times 10^{-5}$ ind.$^{-1}$ day$^{-1}$	(b)
	$\gamma _i$	Recovery rate in Patch *i*	0.25 day$^{-1}$	(a)
	$\delta _i$	Death rate due to disease in Patch *i*	$5.0\times 10^{-4}$ day$^{-1}$	(a)
	$\xi _i$	Shedding rate of pathogen in Patch *i*	$7.56\times 10^{-3}$ day$^{-1}$	(a)
	$\nu _i$	Decay rate of pathogen in Patch *i*	$7.56\times 10^{-3}$ day$^{-1}$	(a)
Movement	$m_1$	Movement rate of healthy individuals from Patch 1 to Patch 2	$5\times 10^{-4}$ day$^{-1}$	(b)
	$m_2$	Movementrate of healthy individuals from Patch 2 to Patch 1	$5\times 10^{-4}$ day$^{-1}$	(b)
	$\rho _1$	Movement rate of pathogen in water from Patch 1 to Patch 2	$1.25\times 10^{-3}$ day$^{-1}$	(b)
	$\rho _2$	Movement rate of pathogen in water out of Patch 2	$1.25\times 10^{-3}$ day$^{-1}$	(b)
Control	$b_i$	Cost per new case in Patch *i*	1 ind.$^{-1}$	(c)
parameters	$A_i$	Cost per vaccination in Patch *i*	0.125 ind.$^{-1}$	(c)
	$B_i$	Cost of water sanitation in Patch *i*	0.0125 ind.$^{-1}$	(c)
	$\epsilon _i$	Nonlinear cost of vaccination in Patch *i*	$1\times 10^{4}$	(c)
	$\eta _i$	Nonlinearcost of water sanitation in Patch *i*	100	(c)
	$u_{i,max}$	Maximum daily water sanitation rate in Patch *i*	0.4 ind.$^{-1}$	(c)
	$v_{i,max}$	Maximum daily vaccination rate in Patch *i*	0.015 ind.$^{-1}$	(a)
	$T_0$	Time until start of control measures	60 days	(c)

Movement parameters were chosen to be realistic, symmetric between patches, and to produce epidemics on a timescale relevant for the control measures considered. Cost coefficients were chosen so that vaccination is one-eighth the cost of a cholera case and sanitation is one-tenth the cost of vaccination. Nonlinear costs were chosen to ensure convergence of the optimal control scheme. Bounds were selected to represent plausible upper limits to control measures of $1.5\%$ of individuals vaccinated per day and $40\%$ reduction in transmission rate from contaminated water due to sanitation. Variations in the cost coefficient parameters are further explored in Sect. 3.3. Parameters were adapted from (a) Kelly et al. (2016) and Tuite et al. (2011), (b) modified from values in those articles, or (c) assigned here in order to get appropriate outbreak simulations and control curves

**Fig. 3:**
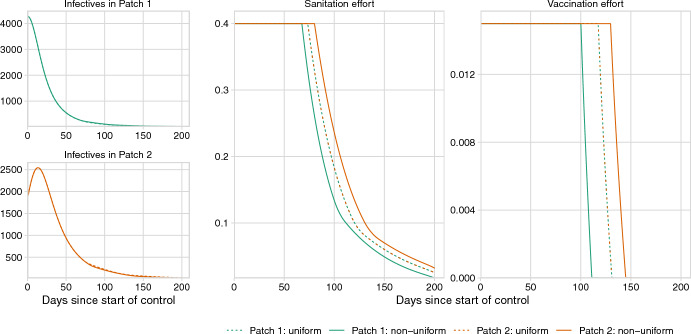
Numerical simulation results for the cholera model, including the numbers of infectious individuals in Patch 1 and Patch 2, and the optimal levels of sanitation and vaccination over time. Across all panels, line color represents the patch number and line type represents the control policy. Each patch begins with 100,000 susceptible individuals with 100 infectious individuals moved to the infectious compartment in Patch 1 to initialize the outbreak

Figure 3 shows the numbers of infectives and the intensity of controls under an optimal control scenario for both non-uniform and uniform policies. The optimal vaccination and sanitation controls eventually decrease the number of infectives in each patch to zero, requiring less control over time. While the number of infectives is also expected to decrease to zero under no control (due to susceptible depletion, see Supplementary Figure D.9), implementation of the optimal control reduces cumulative cases across both patches by up to 62% (56,381 cases with non-uniform control vs. 148,403 cases without any control). Even given the implementation of control, there is an initial increase in infectives in Patch 2 after the onset of controls, due to the flow of infectious agents in the water from Patch 1 to Patch 2.

For both uniform and non-uniform approaches, the optimal sanitation rates remain at their maximum values for approximately 75 days. Although sanitation effort does approach zero, it does not reach zero within the 200 day study period. Vaccination effort remains at its maximum value for more than 100 days and reduces to zero before day 150. In the scenario we consider, the uniform optimal control falls between the patch-specific (i.e., non-uniform) controls. For example, in Fig. 3, the optimal sanitation and vaccination rates in the uniform case are less than the non-uniform control for Patch 2 (orange curves) and greater than the non-uniform control for Patch 1 (green curves).

**Fig. 4:**
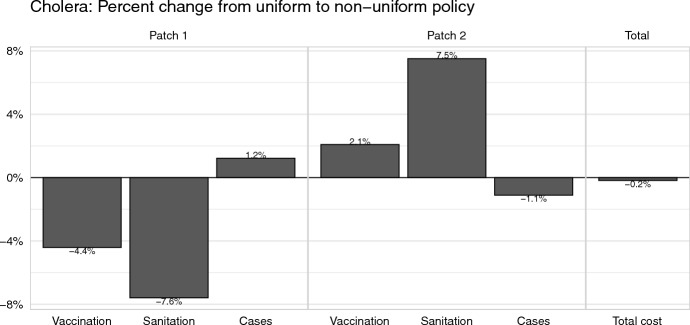
The effect of switching from a uniform to a non-uniform policy for the cholera model. Bars show the percent change in the number of vaccines distributed, amount of water sanitized, number of cholera cases in Patch 1 and 2, as well as the overall cost. Percent change is calculated as $(v_\text {non-uniform}-v_\text {uniform})/v_\text {uniform}$ for each value of interest *v* (i.e., vaccination, sanitation, cases, total cost). Only cholera cases which occurred after the onset of control (day 0) are counted

We measured the effect of switching from a uniform to a non-uniform policy on the number of vaccinations, amount of water sanitized, number of cholera cases, as well as the total cost (Fig. 4). Although the differences between the uniform and non-uniform controls do not significantly change the total cost, the implementation of a uniform control policy leads to substantial shifts in the relative distribution of resources and epidemiological burden across the two patches. Under both policies, the majority of cases and deaths occur in Patch 2 compared to Patch 1 during the control window (Table 2). More resources are allocated to Patch 2 under the non-uniform policy in comparison to the uniform policy, leading to a 2.1% increase in vaccination effort and 7.5% increase in sanitation effort (Fig. 4 and Supplementary Table C.4). These additional resources decrease cases in Patch 2 by 1.1% compared to the uniform policy. The shift to a non-uniform policy, however, decreases the resources allocated to Patch 1, decreasing vaccination by 4.4% and sanitation by 7.6%, leading to a 1.2% increase in cases.

**Table 2:** Case and control totals from the simulations of the cholera and Ebola models under non-uniform and uniform policies after the start of controls (60 days for cholera and 150 days for Ebola)

Model	Control type	Patch	Cases	Deaths	Vaccinations	Second control
Cholera	Non-uniform	1	26,390	61	44,069	41
	Non-uniform	2	29,972	63	58,735	47
	Uniform	1	26,073	61	46,103	44
	Uniform	2	30,309	64	57,535	44
Ebola	Non-uniform	1	5737	1402	55,199	3192
	Non-uniform	2	1267	154	57,688	220
	Uniform	1	5834	1425	53,012	3,108
	Uniform	2	1085	133	61,492	255

The second control for cholera gives the (scaled) volume of contaminated water that was sanitized over the simulation period. The second control for Ebola gives the additional hospitalizations due to the implementation of control over the simulation period

### Ebola virus disease

Our numerical simulations use parameters from Burton et al. (2021) and Blackwood and Childs (2016). Using the same methods as for cholera, we calculated the basic reproduction number of the two-patch Ebola system to ensure that our simulations produced realistic outbreak sizes. For the full derivation and description of the basic reproduction number, see “Appendix A.2”. With the parameter values in Table 3, we obtain ${\mathcal {R}}_0= 1.7$ which is in-line with previous estimates of ${\mathcal {R}}_0$ for Ebola (Getz et al. 2019).

Similar to cholera, we assume each patch has a population of 100,000 individuals, an outbreak begins in Patch 1, and controls are implemented after 150 days. In this case, we ran our model without control from in an initially naive population with 10 infected individuals in Patch 1 (i.e., $S_1(0) = 99{,}990$, $S_2(0) = 100{,}000$, $I_1(0) = 10$, $I_2(0) = 0$, $E_1(0) = E_2(0) = 0$, $D_1(0) = D_2(0) = 0$, $H_1(0) = H_2(0) = 0$, and $R_1(0) = R_2(0) = 0$). Then, we use the size of each compartment at day 150 as initial conditions for all optimal control analyses ($S_1(150) = 89,700$, $S_2(150) = 99{,}428$, $E_1(150) = 2723$, $E_2(150) = 153$, $I_1(150) = 696$, $I_2(150) = 38$, $H_1(150) = 834$, $H_2(150) = 42$, $D_1(150) = 66$, $D_2(150) = 3$, $R_1(150) = 5382$, and $R_2(150) = 282$). During this period, there were 10,871 cases and 69 deaths across both patches.

Using rough estimates of the cost of vaccination described in Bartsch et al. (2015) and UNICEF (2021), we set the linear cost of vaccination, $A_1$ and $A_2$, to 0.01, or 1% of the cost of cases. We assume it is possible to vaccinate at most 1.5% of the population per day, i.e., $v_1(t) = v_2(t) = 0.015$. Further, we assume that increasing the hospitalization rate is ten times more expensive than vaccination and set $B_1$ and $B_2$ as 0.1, or 10% of the cost of cases. Both $u_1(t)$ and $u_2(t)$ have an upper bound of 0.5, meaning that the hospitalization rate can increase by at most 50%. Baseline parameters are summarized in Table 3.

**Table 3:** Symbols, descriptions and values of the baseline Ebola model parameters

		Description	Value	Reference
Disease	$\mu _i$	Natural birth/death rate in Patch *i*	$5.5\times 10^{-5}$ day$^{-1}$	(d)
dynamics	$\beta _{Ii}$	Transmission rate from contact with infectious in Patch *i*	$2.94\times 10^{-6}$ ind.$^{-1}$ day$^{-1}$	(a)
	$\beta _{Di}$	Transmission rate from contact with corpse in Patch *i*	$2.94\times 10^{-5}$ ind.$^{-1}$ day$^{-1}$	(a)
	$\alpha _i$	Incubation rate in Patch *i*	0.1 day$^{-1}$	(c)
	$\varphi _i$	Hospitalization rate of infectious in Patch *i*	0.236 day$^{-1}$	(b)
	$\gamma _{Ii}$	Recovery rate of infectious in Patch *i*	0.1 day$^{-1}$	(c)
	$\gamma _{Hi}$	Recovery rate of hospitalized in Patch *i*	0.154 day$^{-1}$	(b)
	$\delta _{Ii}$	Death rate of infectious due to disease in Patch *i*	0.024 day$^{-1}$	(b)
	$\delta _{Hi}$	Deathrate of hospitalized due to disease in Patch *i*	0.01 day$^{-1}$	(b)
	$\xi _i$	Decay rate of corpses in Patch *i*	0.222 day$^{-1}$	(b)
Movement	$m_1$	Movement rate of healthy individuals from Patch 1 to Patch 2	$5\times 10^{-4}$ day$^{-1}$	(d)
	$m_2$	Movement rate of healthy individuals from Patch 2 to Patch 1	$5\times 10^{-4}$ day$^{-1}$	(d)
Control	$b_i$	Cost per new case in Patch *i*	1 ind.$^{-1}$	(d)
parameters	$A_i$	Cost per vaccination in Patch *i*	.01 ind.$^{-1}$	(d)
	$B_i$	Cost per hospitalization in Patch *i*	0.1 ind.$^{-1}$	(d)
	$\epsilon _i$	Nonlinear cost of vaccination in Patch *i*	$5\times 10^{4}$	(d)
	$\eta _i$	Nonlinear cost of hospitalization in Patch *i*	5	(d)
	$u_{i,max}$	Maximum hospitalization rate in Patch *i*	0.5 day$^{-1}$	(d)
	$v_{i,max}$	Maximum daily vaccination rate in Patch *i*	0.015 day$^{-1}$	(d)
	$T_0$	Time until start of control measures	100 days	(d)

The values for $\beta _{Ii}$ and $\beta _{Di}$ were estimated by assuming that ${\mathcal {R}}_0=1.7$ (Getz et al. 2019) and that transmission from contact with dead bodies is ten times more transmissible than contact with infectious individuals ($\beta _{Di}=10\beta _{Ii}$). All other diseases dynamic parameter values were obtained from Burton et al. (2021) and Blackwood and Childs (2016). Movement parameters were chosen to be realistic, symmetric between patches, and to produce epidemics on a timescale relevant for the control measures considered. Cost coefficients were chosen so that hospitalization is one-tenth the cost of an Ebola case and vaccination is one-tenth the cost of hospitalization. Nonlinear costs were chosen to ensure convergence of the optimal control scheme. Bounds were selected to represent plausible upper limits to control measures of $1.5\%$ of individuals vaccinated per day and a 50% increase in hospitalization rate. Variations in the cost coefficient parameters are further explored in Sect. 3.3. Parameter values were obtained from (a) Getz et al. (2019), (b) Burton et al. (2021), (c) Blackwood and Childs (2016), or (d) assigned here in order to get appropriate outbreak simulations and control curves

**Fig. 5:**
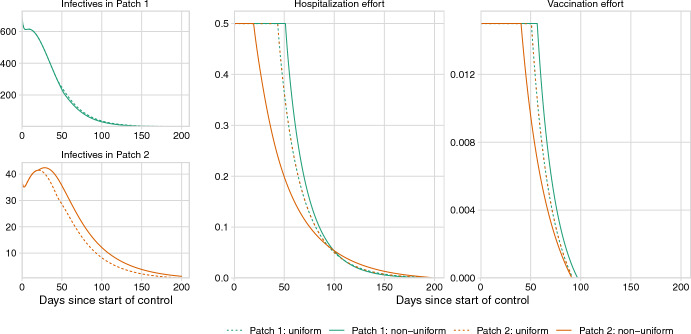
Numerical simulation results for the Ebola model, including the numbers of infectious individuals in Patch 1 and Patch 2, and the optimal levels of hospitalization and vaccination over time. Across all panels, line color represents the patch number and line type represents the control policy. Each patch begins with 100,000 susceptible individuals with 10 infectious individuals moved to the infectious compartment in Patch 1 to initialize the outbreak

Similar to the cholera model, the optimal control of Ebola decreases the number of infectives in each patch, requiring less control over time (Fig. 5); however, some level of control is necessary to bring the number of infectives to zero within the simulation period of 200 days (see Supplementary Figure D.10). For both uniform and non-uniform approaches, the optimal hospitalization and vaccination rates remain at their maximum values for approximately 50 days after which they decrease to 0 before day 200 for hospitalization and day 100 for vaccination. Like in the case of cholera, the uniform optimal control falls between the patch-specific (i.e., non-uniform) controls (Fig. 5). However, in contrast to cholera, Patch 1 (the “source” patch) receives more resources than Patch 2 for the majority of the control period. Although, after 109 days, Patch 2 is allocated more hospitalization effort than Patch 1.

**Fig. 6:**
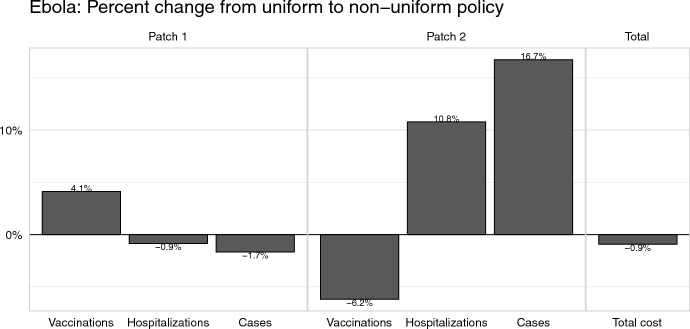
The effect of switching from a uniform to a non-uniform policy for the Ebola model. Bars show the percent change in the number of vaccines distributed, number of individuals hospitalized and number of Ebola cases in Patch 1 and 2, as well as the overall cost. Percent change is calculated as $(v_\text {non-uniform}-v_\text {uniform})/v_\text {uniform}$ for each value of interest *v* (i.e., vaccination, hospitalization, cases, total cost). Only Ebola cases which occurred after the onset of control (day 0) are counted

Like we saw with the results for cholera, the total cost is not significantly different between uniform and non-uniform controls, however, control levels and epidemiological burden differ across the two policies (Fig. 6). Specifically, Patch 1 receives 4.1% more vaccination resources under the non-uniform policy in comparison to the uniform policy. Hospitalization effort, however, increases 10.8% in Patch 2 when switching to a non-uniform policy from a uniform one. This case, where Patch 1 receives more vaccination but Patch 2 receives more hospitalization, is qualitatively different from the cholera model, where Patch 2 receives more of both resources. The change in resource distribution from uniform to non-uniform decreases cases in Patch 1 by 1.7%, but increases cases in Patch 2 by 16.7%.

### The effect of changing cost and movement parameters

We next considered whether, in either model, our results are sensitive to the choice of the control costs and movement parameters. In particular, we consider the effect of asymmetry in the patch-specific costs of control and movement. To do this, we increased parameters by an order of magnitude and observed the changes in the control trajectories for each model and each patch. We omit several cases that did not show interesting shifts in control trajectories: for cholera, increasing the cost of sanitation in Patch 2 and, for Ebola, increasing the costs of either of the controls in either patch, and changes in movement parameters (see Supplementary Figure D.11).

**Fig. 7:**
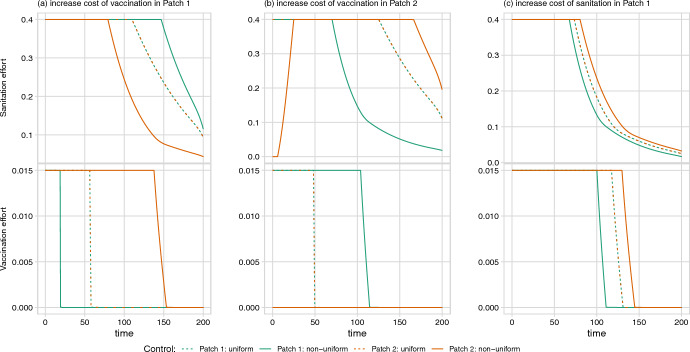
The effect of increasing cost parameters on the optimal control trajectories of the cholera model. Cost parameters are increased by one order of magnitude from baseline: **a** increasing linear cost of vaccination in Patch 1 from $A_1 = 0.125$ to 1.25; **b** increasing linear cost of vaccination in Patch 2 from $A_2 = 0.125$ to 1.25; and **c** increasing linear cost of sanitation in Patch 1 from $B_1 = 0.0125$ to 0.125. The case where the cost of sanitation is increased in Patch 2 is not shown as it is substantially similar to (**c**). Total cost of vaccination and sanitation under each scenario is (**a**) non-uniform: 33,465 and uniform: 53,473, **b** non-uniform: 9,881 and uniform: 60,230, and **c** non-uniform: 16,308 and uniform: 16,419 (compared to the baseline case, with cost of non-uniform: 16,305 and uniform: 16,416)

In the cholera model, increasing the vaccination cost parameters can substantially change the trajectories of all controls (Fig. 7). For example, increasing the cost of vaccination in Patch 1 means fewer vaccines are distributed to Patch 1 in both the uniform and non-uniform cases (Fig. 7a, second row), which is compensated for by increased sanitation effort. Unlike in the baseline case, where Patch 2 receives more sanitation and vaccination than Patch 1 (Fig. 3), the optimal non-uniform policy allocates more vaccination to Patch 2 but more sanitation to Patch 1 when the cost of vaccination is higher in Patch 1 (Fig. 7a). Note how, as in the baseline case, the uniform control trajectories of both patches always fall between the corresponding non-uniform control trajectories.

Increasing the cost of vaccination in Patch 2 leads to substantial qualitative differences in control trajectories (Fig. 7b). Similar to the case where vaccination cost is increased in Patch 1, vaccination effort under the uniform policy is reduced and compensated for by increased levels of sanitation. The optimal non-uniform policy, however, allocates no vaccines to Patch 2. Although the absence of vaccinations in Patch 2 is offset by increased sanitation, the start of sanitation control in Patch 2 is delayed (not rising above 1% until day 7). When compared to the uniform policy, the non-uniform policy provides higher levels of sanitation for Patch 2 and higher levels of vaccination in Patch 1. Compared to increasing the cost of vaccination, the optimal control results are much less sensitive to increases in the cost of sanitation, either in Patch 1 (Fig. 7c) or Patch 2 (not shown). Under both of these parameterizations, the optimal control results are similar to the baseline case.

**Fig. 8:**
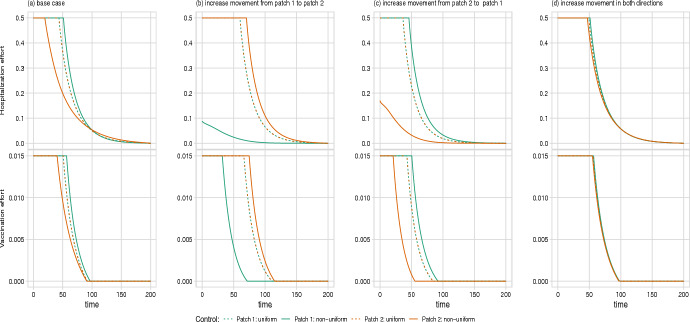
The effect of increasing movement parameters on the optimal control trajectories of the Ebola model. Movement parameters are increased by one order of magnitude: **a** baseline case shown in main text, where movement rate $m_1 = 5\times 10^{-4}$ and $m_2 = 5\times 10^{-4}$; **b** increasing movement rate from Patch 1 to Patch 2, $m_1 = 5\times 10^{-3}$; **c** increasing movement rate from Patch 2 to Patch 1 $m_2 = 5\times 10^{-3}$; **d** increasing movement in both directions, $m_1 = 5\times 10^{-3}$ and $m_2 = 5\times 10^{-3}$

For both diseases, increasing the rate of movement between patches reduces the difference between the non-uniform policy and the uniform policy, as the two populations became more closely linked and act more like a well-mixed population. Asymmetrical increases in movement rates lead to patches of unequal size; this in turn results in an optimal non-uniform control that places increased emphasis on control in the more populous patch (Fig. 8). This result is less pronounced in the cholera model, where the flow of contaminated water from Patch 1 to Patch 2 is a strong driver of dynamics and thus maintains the need for controlling the outbreak in Patch 1, even when Patch 2 has a larger population (Supplementary Figure D.12).

## Discussion

The choice of governance structure is often ignored in mathematical studies of disease management. However, recent work suggests that this choice may be consequential for outbreak suppression (Blackwood et al. 2021). In this paper, we used two case studies to investigate the differences between two control policies of a central manager: “uniform”, where the level of control must be the same in both patches, and “non-uniform”, where the level of control is allowed to vary between the patches. We found that, while the choice of control policy has only a marginal effect on the total cost of control, this choice may exacerbate inequalities between jurisdictions, both in the size of outbreaks and the amount of resources allocated.

It is no surprise that following a non-uniform control policy leads to an overall lower cost of control: the set of all possible uniform control policies is a subset of all possible non-uniform policies. But in both of our case studies, non-uniform control policies led to a small (less than $1\%$) decrease in overall cost when compared to uniform control policies (Figs. 4 and 6). Thus a manager deciding between employing a non-uniform or uniform control policy may not focus entirely on the total cost incurred by each policy to make their choice but instead consider other alternative consequences of their choice.

The distribution of resources and epidemiological burden may change substantially when shifting to a different control policy. Our case studies showed that shifting from a uniform to a non-uniform policy could lead to changes in the outbreak sizes ranging from a $1.2\%$ decrease to a $16.7\%$ increase (Figs. 4 and 6). A stark example of these disparities is found in our Ebola case study. Under a uniform control policy, Patch 2, the patch that is not the initial source of the outbreak, received fewer vaccination and hospitalization resources and thus saw a $16.7\%$ relative increase in the burden of cases when compared to the non-uniform policy (Figs. 5 and 6). In this case, a manager may prefer a uniform policy, which minimizes the disparities between the patches, at the cost of a $0.9\%$ relative increase in total cost.

In our examples, the uniform control policy is always bounded by the non-uniform controls for each patch. Investigating whether this result holds in general or if it is tied to our model formulation or parameterization is an important future direction. From a certain perspective, this result implies that when switching from a uniform to non-uniform policy, one patch will always benefit from the switch in terms of increased resource allocation, reduced number of cases, or both, while the other patch will always be disadvantaged due to the switch. Thus, despite the facts that the uniform policy is more equitable (in the sense of resource allocation) and that the overall total cost is relatively similar between the two policies, there are still meaningful trade-offs that a manager should take into account when choosing a policy.

In addition to inducing disparities between patches, the implementation of a non-uniform policy may come with additional costs that we have not considered explicitly. For example, there may be political costs associated with disparities in the distribution of resources across geopolitical regions. Similarly, there may be increased implementation costs incurred when managing two jurisdictions separately. A manager might also consider additional costs associated with disease-induced mortality, which we did not include in our cost calculations in this study.

The case studies in this paper also illustrate how pathogen transmission modes and the connectivity of patches can alter the optimal allocation of resources for disease control. In the Ebola model (Fig. 5), Patch 1, the source of the outbreak, receives more resources for the majority of the simulation time. On the other hand, in the cholera model, Patch 2 receives more resources under non-uniform control despite the outbreak originating in Patch 1 (Fig. 3). Whereas an Ebola outbreak might decrease migration between the patches (because infected individuals do not travel), a cholera outbreak may increase transmission to Patch 2 because of shedding into the water that then flows downstream to Patch 2. These results suggest that understanding and accounting for connectivity between patches is essential for effective management of infectious diseases.

For our baseline cases, we assumed that the patches in our model were largely symmetric (including transmission biology, implementation cost, and movement of individuals). However, in practice, asymmetries between patches are likely to be the norm rather than the exception. We investigated two potential asymmetries, specifically the cases where the implementation costs of the controls are elevated in one patch and where the movement of healthy individuals is greater in one direction. In most of the cases we considered, optimal control strategies are insensitive to a single-patch increase in implementation cost. However, increasing the cost of vaccination in one patch within the cholera model changed the shape of the control functions qualitatively. As expected, the patch with the higher vaccination cost received fewer vaccinations, which is compensated for by an increase in sanitation (Fig. 7). Changing the movement parameters had a qualitative effect on the optimal control strategies only when the migration was asymmetric and the primary mode of transmission was direct (as in Ebola) suggesting that a policy of restricting immigration could have unintended consequences. Further investigation into the effects of patch-specific differences is warranted to generalize this work.

In this study, we only considered the policies of a central manager allocating resources across two patches. However, governance across regions is often decentralized and jurisdictions act independently or semi-independently (e.g., US state-level stay-at-home orders during the COVID-19 pandemic Moreland et al. 2020). Separate jurisdictions may enact different types and levels of responses and initiate their responses at different time points. Modeling a more realistic, decentralized governance structure introduces additional complications because the optimization of the costs within a single patch would require knowledge of the actions taken in other patches. To address such a challenge, other studies have assumed that patches ignore one another (Blackwood et al. 2021) or have used game theory to model between-patch dynamics (Sanchirico et al. 2021).

This novel application of optimal control theory to investigate governance structures for infectious disease management yielded several insights. Our case studies suggest that central managers who care only about minimizing total cost should use a non-uniform control policy. However, the relative cost savings of following a non-uniform policy over a uniform one are minimal. Thus a manager motivated to maintain an equal resource allocation between the patches will prefer a uniform control policy. But that is not to say a uniform control policy is always equitable: choosing one policy over another inevitably introduces disparities in the outbreak sizes and resource allocations in the patches. Responsible management of an outbreak across two patches therefore requires a holistic perspective of the impacts of policy choice on both patches, as well as a firm understanding of the underlying dynamics of disease transmission. Further investigation into the interplay between governance structure and optimal control of infectious disease outbreaks is important for continuing to improve the efficacy and equity of infectious disease management.

### Supplementary Information

Below is the link to the electronic supplementary material.

Supplementary file 1 (pdf 1082 KB)

## Data Availability

The code used to generate the results in this article can be found at https://github.com/eahowerton/governance-and-disease-control and archived at https://zenodo.org/badge/latestdoi/528556355.

## Appendix A: Details of basic reproduction number calculations

### Appendix A.1: Cholera

We define the vector $x = (I_1, I_2, W_1, W_2, S_1, S_2, R_1, R_2)^T$. There is a unique disease free equilibrium given by $x_0 = (0, 0, 0, 0, N_{1,0}, N_{2,0}, 0, 0)^T$ where $N_{i,0}$ represents the initial population in Patch *i*. For each patch there are only two infectious compartments: infected humans ($I_i$) and the water reservoir ($W_i$), but new infections only occur in the infected humans compartment. Let ${\mathscr {F}}(x)$ be the rate of new infections, and let ${\mathscr {V}}(x)$ be the net transfer for all other transitions. Under this condition, the system (1) can be written as $x' = {\mathscr {F}}(x) - {\mathscr {V}}(x)$. In computing the next generation matrix, we define

$$\begin{aligned} {\mathcal {R}}_{Ii}&= \dfrac{\beta _{Ii}N_{i,0}}{\gamma _i+\mu _i+\delta _i}\\ {\mathcal {R}}_{Wi}&= \dfrac{\beta _{Wi}N_{i,0}\xi _i}{(\gamma _i+\mu _i+\delta _i)(\xi _i+\rho _i)}. \end{aligned}$$

These expressions denote the new infections in Patch *i* resulting from contact with an infected individual $({\mathcal {R}}_{Ii})$ and the new infections resulting from contact with contaminants in the water source $({\mathcal {R}}_{Wi})$. Thus the basic reproductive number for Patch *i* as an isolated system is ${\mathcal {R}}_{Ii}+{\mathcal {R}}_{Wi}$. Because we do not allow infected individuals to move between patches, the next generation matrix is block-upper triangular (with a zero matrix in the lower-diagonal block). The principal $2\times 2$ submatrix has the form

$$\begin{aligned} \begin{bmatrix} {\mathcal {R}}_{I1}+{\mathcal {R}}_{W1} &{} 0 \\ \left( \dfrac{\rho _1}{\xi _1+\rho _1}\right) \left( \dfrac{\xi _1(\mu _2+\gamma _2+\delta _2)}{\xi _2(\mu _1+\gamma _1+\delta +1)}\right) &{} {\mathcal {R}}_{I2} + {\mathcal {R}}_{W2} \end{bmatrix} \end{aligned}$$

The basic reproduction number of our system is the spectral radius of this $2\times 2$ submatrix. Thus, in the absence of movement among infected individuals, the basic reproduction number is the larger of the two diagonal entries, i.e., the patch-specific reproduction numbers.

### Appendix A.2: Ebola

To compute the reproduction number of the Ebola model, we proceed in a similar fashion using the next generation matrix method. We define the vector $x = (E_1, E_2, I_1, I_2, D_1, D_2, S_1, S_2, H_1, H_2, R_1, R_2)^T$ and write the state equations as $x' ={\mathscr {F}}(x) -{\mathscr {V}}(x)$ where ${\mathscr {F}}(x)$ contains terms representing new infections and ${\mathscr {V}}(x)$ contains all other transition terms. In this model, new infections appear in the exposed class ($E_i$) as a result of direct contact with an infected individual ($I_i$) or through contact with a recently deceased person ($D_i$). Because the exposed class is permitted to move between patches, an individual from Patch 1 can cause a new infection in Patch 2 (and vice versa). The next generation matrix exhibits this structure. For the two patches, let ${\mathcal {R}}_{ij}$ be a type reproduction number representing the expected number of new infections in Patch *j* due to an initial infective in Patch *i*. The basic reproduction number is then given by

$$\begin{aligned} {\mathcal {R}}_0 = \dfrac{1}{2}({\mathcal {R}}_{11} + {\mathcal {R}}_{22}) + \dfrac{1}{2}\sqrt{({\mathcal {R}}_{11} + {\mathcal {R}}_{22})^2 - 4({\mathcal {R}}_{11}{\mathcal {R}}_{22} - {\mathcal {R}}_{12}{\mathcal {R}}_{21})}. \end{aligned}$$

## Appendix B: Details of optimality systems

### Appendix B.1: Cholera

First, we describe the characterization of the optimal control for the non-uniform policy, in which the controls in each patch may be different. Let our state variables be defined as solutions of the system of differential equations in (1). Our optimal control problem consists of finding a control vector $(u_1^*, v_1^*, u_2^*, v_2^*)$ from the control set (3) that satisfies

$$\begin{aligned} J(u_1^*, v_1^*, u_2^*, v_2^*) = \min _U J(u_1, v_1, u_2, v_2), \end{aligned}$$

where $J(u_1, v_1, u_2, v_2)$ is the objective functional given in (2). We append the right hand sides of our state differential equations to the integrand of the objective functional with adjoint functions $\lambda = [\lambda _1, \lambda _2, \dots , \lambda _{8}]$ by defining the Hamiltonian in (15).

15 $$\begin{aligned} \begin{aligned} H&= b_1(\beta _{I1}S_1I_1 + (1 - u_1)\beta _{W1}S_1W_1) + A_1v_1S_1 + \epsilon _1v^2_1 + B_1u_1 + \eta _1u_1^2 \\&\quad + b_2(\beta _{I2}S_2I_2 + (1 - u_2)\beta _{W2}S_2 W_2) + A_2v_2S_2 + \epsilon _2v^2_2 + B_2u_2 + \eta _2u_2^2 \\&\quad + \lambda _1(\mu _1(S_1 + I_1 + R_1) - \beta _{I1}S_1I_1 - (1 - u_1)\beta _{W1}S_1W_1 - \mu _1S_1 - v_1S_1 \\&\quad - m_1S_1 + m_2S_2) + \lambda _2(\beta _{I1}S_1I_1 + (1 - u_1)\beta _{W1}S_1W_1 - (\gamma _1 + \mu _1 + \delta _1)I_1 ) \\&\quad + \lambda _3(\gamma _1I_1 - \mu _1R_1 + v_1S_1 - m_1R_1 + m_2R_2) \\&\quad + \lambda _4(\xi _1I_1 - \xi _1W_1 - \rho _1W_1) + \lambda _5(\mu _2(S_2 + I_2 + R_2) - \beta _{I2}S_2I_2\\&\quad - (1 - u_2)\beta _{W2}S_2W_2 - \mu _2S_2 - v_2S_2 + m_1S_1 - m_2S_2) \\&\quad + \lambda _6(\beta _{I2}S_2I_2 + (1 - u_2)\beta _{W2}S_2W_2 - (\gamma _2 + \mu _2 + \delta _2)I_2 ) \\&\quad + \lambda _7(\gamma _2I_2 - \mu _2R_2 + v_2S_2 + m_1R_1 - m_2R_2) \\&\quad + \lambda _8(\xi _2I_2 - \xi _2W_2 + \rho _1W_1 - \rho _2W_2) \end{aligned} \nonumber \\ \end{aligned}$$

Pontryagin’s Maximum Principle states that if $(u_1^*, v_1^*, u_2^*, v_2^*)$ and the corresponding states minimize the objective functional (2), then $(u_1^*, v_1^*, u_2^*, v_2^*)$ minimizes the Hamiltonian, *H*, in (15) with respect to the controls at each time. Thus, on the interior of the control set, we can find $(u_1^*, v_1^*, u_2^*, v_2^*)$ by solving the optimality conditions

$$\begin{aligned} \frac{\partial H}{\partial u_i} = 0 \quad \text {and} \quad \frac{\partial H}{\partial v_i} = 0, \quad i = 1,2, \end{aligned}$$

for the controls $u_1^*$, $v_1^*$, $u_2^*$ and $v_2^*$:

16 $$\begin{aligned} \frac{\partial H}{\partial u_1}= & {} -b_1\beta _{W1}S_1W_1 + B_1 + 2\eta _1u_1^* + \lambda _1\beta _{W1}S_1W_1 - \lambda _2\beta _{W1}S_1W_1 = 0 \nonumber \\ \implies u_1^*= & {} \frac{b_1\beta _{W1}S_1W_1 - B_1 - \lambda _1\beta _{W1}S_1W_1 + \lambda _2\beta _{W1}S_1W_1}{2\eta _1}\nonumber \\ \frac{\partial H}{\partial u_2}= & {} -b_2\beta _{W2}S_2W_2 + B_2 + 2\eta _2u_2^* + \lambda _5\beta _{W2}S_2W_2 - \lambda _6\beta _{W2}S_2W_2 = 0 \nonumber \\ \implies u_2^*= & {} \frac{b_2\beta _{W2}S_2W_2 - B_2 - \lambda _5\beta _{W2}S_2W_2 + \lambda _6\beta _{W2}S_2W_2}{2\eta _2}\nonumber \\ \frac{\partial H}{\partial v_1}= & {} A_1S_1 + 2\epsilon _1v_1^* - \lambda _1S_1 + \lambda _3S_1 = 0 \nonumber \\ \implies v_1^*= & {} \frac{\lambda _1S_1 - A_1S_1 - \lambda _3S_1}{2\epsilon _1} \nonumber \\ \frac{\partial H}{\partial v_2}= & {} A_2S_2 + 2\epsilon _2v_2^* - \lambda _5S_2 + \lambda _7S_2 = 0 \nonumber \\ \implies v_2^*= & {} \frac{\lambda _5S_2 - A_2S_2 - \lambda _7S_2}{2\epsilon _2}. \end{aligned}$$

The optimal control characterizations (5) are found by taking into account the bounds on the control representations in (16). Since $\eta _i > 0$ and $\epsilon _i > 0$ for $i = 1,2$, we have the appropriate convexity,

$$\begin{aligned} \frac{\partial ^2 H}{\partial u_i^2} = 2\eta _i> 0 \quad \text {and} \quad \frac{\partial ^2 H}{\partial v_i^2} = 2\epsilon _i > 0, \quad i = 1,2, \end{aligned}$$

which ensures that the optimal control $(u_1^*, v_1^*, u_2^*, v_2^*)$ is in fact a minimum.

The optimality system consists of the characterization for the optimal control $(u_1^*, v_1^*, u_2^*, v_2^*)$ in (5) together with the state and adjoint equations (and their boundary conditions). To form the optimality system, we need the adjoint differential equations:

$$\begin{aligned} \lambda _1'&= -\frac{\partial H}{\partial S_1} = -[b_1(\beta _{I1}I_1 + (1 - u_1)\beta _{W1}W_1) + A_1v_1 \\&\hspace{10pt} + \lambda _1(\mu _1 - \beta _{I1}I_1 - (1 - u_1)\beta _{W1}W_1 - \mu _1 - v_1 - m_1)\\&\hspace{10pt} + \lambda _2(\beta _{I1}I_1 + (1 - u_1)\beta _{W1}W_1) + \lambda _3(v_1) + \lambda _5(m_1)] \\ \lambda _2'&= -\frac{\partial H}{\partial I_1} = -[b_1\beta _{I1}S_1 + \lambda _1(\mu _1 - \beta _{I1}S_1) + \lambda _2(\beta _{I1}S_1 \\&\hspace{10pt} - (\gamma _1 + \mu _1 + \delta _1 )) + \lambda _3(\gamma _1) + \lambda _4(\xi _1) ] \\ \lambda _3'&= -\frac{\partial H}{\partial R_1} = -[\lambda _1(\mu _1) - \lambda _3(\mu _1 + m_1) + \lambda _7(m_1)] \\ \lambda _4'&= -\frac{\partial H}{\partial W_1} = -[b_1(1 - u_1)\beta _{W1}S_1 - \lambda _1((1 - u_1)\beta _{W1}S_1)\\&\hspace{10pt} + \lambda _2((1 - u_1)\beta _{W1}S_1) - \lambda _4(\xi _1 + \rho _1) + \lambda _8(\rho _1)] \\ \lambda _5'&= -\frac{\partial H}{\partial S_2} = -[b_2(\beta _{I2}I_2 + (1 - u_2)\beta _{W2}W_2) + A_2v_2 + \lambda _1(m_2) \\&\hspace{10pt} + \lambda _5(\mu _2 - \beta _{I2}I_2 - (1 - u_2)\beta _{W2}W_2 - \mu _2 - v_2 - m_2)\\&\hspace{10pt} + \lambda _6(\beta _{I2}I_2 + (1 - u_2)\beta _{W2}W_2) + \lambda _7(v_2)] \\ \lambda _6'&= -\frac{\partial H}{\partial I_2} = -[b_2\beta _{I2}S_2 + \lambda _5(\mu _2 - \beta _{I2}S_2) + \lambda _6(\beta _{I2}S_2 - (\gamma _2 + \mu _2 + \delta _2 )) \\&\hspace{10pt}+ \lambda _7(\gamma _2) + \lambda _8(\xi _2)] \\ \lambda _7'&= -\frac{\partial H}{\partial R_2} = -[\lambda _3(m_2) + \lambda _5(\mu _2) - \lambda _7(\mu _2 + m_2)] \\ \lambda _8'&= -\frac{\partial H}{\partial W_2} = -[b_2(1 - u_2)\beta _{W2}S_2 - \lambda _5(1 - u_2)\beta _{W2}S_2\\&\hspace{10pt} + \lambda _6(1 - u_2)\beta _{W2}S_2 - \lambda _8(\xi _2 + \rho _2)] \\ \lambda _j(T)&= 0, \quad j = 1,2,\dots ,8. \end{aligned}$$

Next, we describe the characterization of the optimal control for the uniform policy, where each patch responds to its outbreak using the same controls as the other patch. We let $u_1 = u_2 = u$ and $v_1 = v_2 = v$. Our optimal control problem consists of finding a control vector $(u^*, v^*)$ from the control set (6) that minimizes the objective functional (2). We find $(u^*, v^*)$ on the interior of the control set, by solving the optimality conditions

$$\begin{aligned} \frac{\partial H}{\partial u} = 0 \quad \text {and} \quad \frac{\partial H}{\partial v} = 0, \end{aligned}$$

for the controls $u^*$ and $v^*$:

17 $$\begin{aligned} \begin{aligned} \frac{\partial H}{\partial u_1}&= -b_1\beta _{W1}S_1W_1 + B_1 + 2\eta _1u_1^* + \lambda _1\beta _{W1}S_1W_1 - \lambda _2\beta _{W1}S_1W_1 \\&\quad - b_2\beta _{W2}S_2W_2 + B_2 + 2\eta _2u_2^* + \lambda _5\beta _{W2}S_2W_2 - \lambda _6\beta _{W2}S_2W_2 = 0 \\ \implies u^*&= {{\frac{\begin{array}{c}b_1\beta _{W1}S_1W_1 - B_1 - \lambda _1\beta _{W1}S_1W_1 + \lambda _2\beta _{W1}S_1W_1 + b_2\beta _{W2}S_2W_2\\ - B_2 - \lambda _5\beta _{W2}S_2W_2 + \lambda _6\beta _{W2}S_2W_2\end{array}}{2(\eta _1 + \eta _2)}}} \\ \frac{\partial H}{\partial v_1}&= A_1S_1 + 2\epsilon _1v_1^* - \lambda _1S_1 + \lambda _3S_1 + A_2S_2 + 2\epsilon _2v_2^* - \lambda _5S_2 + \lambda _7S_2= 0 \\ \implies v^*&= \frac{\lambda _1S_1 - A_1S_1 - \lambda _3S_1 + \lambda _5S_2 - A_2S_2 - \lambda _7S_2}{2(\epsilon _1 + \epsilon _2)}. \end{aligned}\nonumber \\ \end{aligned}$$

The optimal control characterizations (7) are found by taking into account the bounds on the control representations in (17). Since $\eta _i > 0$ and $\epsilon _i > 0$ for $i = 1,2$, we have the appropriate convexity,

$$\begin{aligned} \frac{\partial ^2 H}{\partial u^2} = 2(\eta _1 + \eta _2)> 0 \quad \text {and} \quad \frac{\partial ^2 H}{\partial v^2} = 2(\epsilon _1 + \epsilon _2) > 0. \end{aligned}$$

### Appendix B.2: Ebola

First, we describe the characterization of the optimal control for the non-uniform policy, in which the controls in each patch may be different. Let our state variables be defined as solutions of the system of differential equations in (8). Our optimal control problem consists of finding a control vector $(u_1^*, v_1^*, u_2^*, v_2^*)$ from the control set (10) that satisfies

$$\begin{aligned} J(u_1^*, v_1^*, u_2^*, v_2^*) = \min _U J(u_1, v_1, u_2, v_2), \end{aligned}$$

where $J(u_1, v_1, u_2, v_2)$ is the objective functional given in (9). We append the right hand sides of our state differential equations to the integrand of the objective functional with adjoint functions $\lambda = [\lambda _1, \lambda _2, \dots , \lambda _{12}]$ by defining the Hamiltonian in (18).

18 $$\begin{aligned} \begin{aligned} H&= b_1(\beta _{I1}S_1I_1 + \beta _{D1}S_1D_1) + A_1v_1(S_1 + E_1) + \epsilon _1v_1^2 + B_1u_1\varphi _1I_1 + \eta _1u_1^2 \\&\quad + b_2(\beta _{I2}S_2I_2 + \beta _{D2}S_2D_2) + A_2v_2(S_2 + E_2) + \epsilon _2v_2^2 + B_2u_2\varphi _2I_2 + \eta _2u_2^2 \\&\quad + \lambda _1(\mu _1N_1 - \beta _{I1}S_1I_1 - \beta _{D1}S_1D_1 - (\mu _1 + v_1 + m_1)S_1 + m_2S_2) \\&\quad + \lambda _2(\beta _{I1}S_1I_1 + \beta _{D1}S_1D_1 - (\mu _1 + \alpha _1 + m_1)E_1 + m_2E_2) \\&\quad + \lambda _3(\alpha _1E_1 - (\mu _1 + \gamma _{I1} + (1 + u_1)\varphi _1 + \delta _{I1} )I_1 ) \\&\quad + \lambda _4((1 + u_1)\varphi _1I_1 - (\mu _1 + \gamma _{H1} + \delta _{H1})H_1) \\&\quad + \lambda _5(\delta _{I1}I_1 - \xi _1D_1) \\&\quad + \lambda _6(v_1S_1 + \gamma _{I1}I_1 + \gamma _{H1}H_1 - (\mu _1 + m_1)R_1 + m_2R_2) \\&\quad + \lambda _7(\mu _2N_2 - \beta _{I2}S_2I_2 - \beta _{D2}S_2D_2 - (\mu _2 + v_2 + m_2)S_2 + m_1S_1) \\&\quad + \lambda _8(\beta _{I2}S_2I_2 + \beta _{D2}S_2D_2 - (\mu _2 + \alpha _2 + m_2)E_2 + m_1E_1) \\&\quad + \lambda _9(\alpha _2E_2 - (\mu _2 + \gamma _{I2} + (1 + u_2)\varphi _2 + \delta _{I2} )I_2 ) \\&\quad + \lambda _{10}((1 + u_2)\varphi _2I_2 - (\mu _2 + \gamma _{H2} + \delta _{H2})H_2) \\&\quad + \lambda _{11}(\delta _{I2}I_2 - \xi _2D_2) \\&\quad + \lambda _{12}(v_2S_2 + \gamma _{I2}I_2 + \gamma _{H2}H_2 - (\mu _2 + m_2)R_2 + m_1R_1) \end{aligned} \nonumber \\ \end{aligned}$$

Pontryagin’s Maximum Principle states that if $(u_1^*, v_1^*, u_2^*, v_2^*)$ and the corresponding states minimize the objective functional (9), then $(u_1^*, v_1^*, u_2^*, v_2^*)$ minimizes the Hamiltonian, *H*, in (18) with respect to the controls at each time. Thus, on the interior of the control set, we can find $(u_1^*, v_1^*, u_2^*, v_2^*)$ by solving the optimality conditions

$$\begin{aligned} \frac{\partial H}{\partial u_i} = 0 \quad \text {and} \quad \frac{\partial H}{\partial v_i} = 0, \quad i = 1,2, \end{aligned}$$

for the controls $u_1^*$, $v_1^*$, $u_2^*$ and $v_2^*$:

19 $$\begin{aligned} \frac{\partial H}{\partial v_1}= & {} A_1(S_1 + E_1) + 2\epsilon _1v_1^* - \lambda _1S_1 + \lambda _6S_1 = 0 \nonumber \\ \implies v_1^*= & {} \frac{-A_1(S_1 + E_1) + \lambda _1S_1 - \lambda _6S_1}{2\epsilon _1} \nonumber \\ \frac{\partial H}{\partial v_2}= & {} A_2(S_2 + E_2) + 2\epsilon _2v_2^* - \lambda _7S_2 + \lambda _{12}S_2 = 0 \nonumber \\ \implies v_2^*= & {} \frac{-A_2(S_2 + E_2) + \lambda _7S_2 - \lambda _{12}S_2}{2\epsilon _2} \nonumber \\ \frac{\partial H}{\partial u_1}= & {} B_1\varphi _1I_1 + 2\eta _1u_1^* - \lambda _3\varphi _1I_1 + \lambda _4\varphi _1I_1 = 0 \nonumber \\ \implies u_1^*= & {} \frac{-B_1\varphi _1I_1 + \lambda _3\varphi _1I_1 - \lambda _4\varphi _1I_1}{2\eta _1} \nonumber \\ \frac{\partial H}{\partial u_2}= & {} B_2\varphi _2I_2 + 2\eta _2u_2^* - \lambda _9\varphi _2I_2 + \lambda _{10}\varphi _2I_2 = 0 \nonumber \\ \implies u_2^*= & {} \frac{-B_2\varphi _2I_2 + \lambda _9\varphi _2I_2 - \lambda _{10}\varphi _2I_2}{2\eta _2}. \end{aligned}$$

The optimal control characterizations (12) are found by taking into account the bounds on the control representations in (19). Since $\eta _i > 0$ and $\epsilon _i > 0$ for $i = 1,2$, we have the appropriate convexity,

$$\begin{aligned} \frac{\partial ^2 H}{\partial u_i^2} = 2\eta _i> 0 \quad \text {and} \quad \frac{\partial ^2 H}{\partial v_i^2} = 2\epsilon _i > 0, \quad i = 1,2, \end{aligned}$$

which ensures that the optimal control $(u_1^*, v_1^*, u_2^*, v_2^*)$ is in fact a minimum.

The optimality system consists of the characterization for the optimal control $(u_1^*, v_1^*, u_2^*, v_2^*)$ in (12) together with the state and adjoint equations. To solve the optimality system, we need the adjoint differential equations:

$$\begin{aligned} \lambda _1'&= -\frac{\partial H}{\partial S_1} = -[b_1(\beta _{I1}I_1 + \beta _{D1}D_1) + A_1v_1 \\&\hspace{10pt} + \lambda _1(\mu _1 -\beta _{I1}I_1 - \beta _{D1}D_1 - \mu _1 - v_1 - m_1) \\&\hspace{10pt} + \lambda _2(\beta _{I1}I_1 + \beta _{D1}D_1) + \lambda _6(v_1) + \lambda _7(m_1)] \\ \lambda _2'&= -\frac{\partial H}{\partial E_1} = -[A_1v_1 + \lambda _1(\mu _1) + \lambda _2(-\mu _1 - \alpha _1 - m_1) + \lambda _3(\alpha _1) + \lambda _8(m_1)] \\ \lambda _3'&= -\frac{\partial H}{\partial I_1} = -[b_1(\beta _{I1}S_1) + B_1u_1\varphi _1 + \lambda _1(\mu _1 - \beta _{I1}S_1) + \lambda _2(\beta _{I1}S_1) \\&\hspace{10pt} + \lambda _3(-(\mu _1 + \gamma _{I1} + (1 + u_1)\varphi _1 + \delta _{I1} )) + \lambda _4((1 + u_1)\varphi _1) \\&\hspace{10pt} + \lambda _5(\delta _{I1}) + \lambda _6(\gamma _{I1}) ] \\ \lambda _4'&= -\frac{\partial H}{\partial H_1} = -[\lambda _1(\mu _1) + \lambda _4(-(\mu _1 + \gamma _{H1} + \delta _{H1})) + \lambda _6(\gamma _{H1})] \\ \lambda _5'&= -\frac{\partial H}{\partial D_1} = -[b_1(\beta _{D1}S_1) + \lambda _1(-\beta _{D1}S_1) + \lambda _2(\beta _{D1}S_1) + \lambda _5(-\xi _1)] \\ \lambda _6'&= -\frac{\partial H}{\partial R_1} = -[\lambda _1(\mu _1) + \lambda _6(-\mu _1 - m_1) + \lambda _{12}(m_1)] \\ \lambda _7'&= -\frac{\partial H}{\partial S_2} = -[b_2(\beta _{I2}I_2 + \beta _{D2}D_2) + A_2v_2 + \lambda _7(\mu _2 -\beta _{I2}I_2\\&\hspace{10pt} - \beta _{D2}D_2 - \mu _2 - v_2 - m_2) \\&\hspace{10pt} + \lambda _8(\beta _{I2}I_2 + \beta _{D2}D_2) + \lambda _{12}(v_2) + \lambda _1(m_2)] \\ \lambda _8'&= -\frac{\partial H}{\partial E_2} = -[A_2v_2 + \lambda _7(\mu _2) + \lambda _8(-\mu _2 - \alpha _2 - m_2) + \lambda _9(\alpha _2) + \lambda _2(m_2)] \\ \lambda _9'&= -\frac{\partial H}{\partial I_2} = -[b_2(\beta _{I2}S_2) + B_2u_2\varphi _2 + \lambda _7(\mu _2 - \beta _{I2}S_2) + \lambda _8(\beta _{I2}S_2) \\&\hspace{10pt} + \lambda _9(-(\mu _2 + \gamma _{I2} + (1 + u_2)\varphi _2 + \delta _{I2} )) + \lambda _{10}((1 + u_2)\varphi _2) + \lambda _{11}(\delta _{I2}) \\&\hspace{10pt} + \lambda _{12}(\gamma _{I2}) ] \\ \lambda _{10}'&= -\frac{\partial H}{\partial H_2} = -[\lambda _7(\mu _2) + \lambda _{10}(-(\mu _2 + \gamma _{H2} + \delta _{H2})) + \lambda _{12}(\gamma _{H2})] \\ \lambda _{11}'&= -\frac{\partial H}{\partial D_2} = -[b_2(\beta _{D2}S_2) + \lambda _7(-\beta _{D2}S_2) + \lambda _8(\beta _{D2}S_2) + \lambda _{11}(-\xi _2)] \\ \lambda _{12}'&= -\frac{\partial H}{\partial R_2} = -[\lambda _7(\mu _2) + \lambda _{12}(-\mu _2 - m_2) + \lambda _6(m_2)] \\ \lambda _j(T)&= 0, \quad j = 1,2,\ldots ,12. \end{aligned}$$

Next, we describe the characterization of the optimal control for the uniform policy, where each patch responds to its outbreak using the same controls as the other patch. We let $u_1 = u_2 = u$ and $v_1 = v_2 = v$. Our optimal control problem consists of finding a control vector $(u^*, v^*)$ from the control set (13) that minimizes the objective functional (9). We find $(u^*, v^*)$, on the interior of the control set, by solving the optimality conditions

$$\begin{aligned} \frac{\partial H}{\partial u} = 0 \quad \text {and} \quad \frac{\partial H}{\partial v} = 0, \end{aligned}$$

for the controls $u^*$ and $v^*$:

20 $$\begin{aligned} \begin{aligned} \frac{\partial H}{\partial v}&= A_1(S_1 + E_1) + 2\epsilon _1v^* - \lambda _1S_1 + \lambda _6S_1 + A_2(S_2 + E_2) + 2\epsilon _2v^*\\&\hspace{10pt} - \lambda _7S_2 + \lambda _{12}S_2 = 0 \\ \implies v^*&= \frac{-A_1(S_1 + E_1) + \lambda _1S_1 - \lambda _6S_1 - A_2(S_2 + E_2) + \lambda _7S_2 - \lambda _{12}S_2}{2(\epsilon _1 + \epsilon _2)} \\ \frac{\partial H}{\partial u}&= B_1\varphi _1I_1 + 2\eta _1u^* - \lambda _3\varphi _1I_1 + \lambda _4\varphi _1I_1 + B_2\varphi _2I_2 + 2\eta _2u^*\\&\hspace{10pt} - \lambda _9\varphi _2I_2 + \lambda _{10}\varphi _2I_2 = 0 \\ \implies u^*&= \frac{-B_1\varphi _1I_1 + \lambda _3\varphi _1I_1 - \lambda _4\varphi _1I_1 - B_2\varphi _2I_2 + \lambda _9\varphi _2I_2 - \lambda _{10}\varphi _2I_2}{2(\eta _1 + \eta _2)}. \end{aligned} \end{aligned}$$

The optimal control characterizations (14) are found by taking into account the bounds on the control representations in (20). Since $\eta _i > 0$ and $\epsilon _i > 0$ for $i = 1,2$, we have the appropriate convexity,

$$\begin{aligned} \frac{\partial ^2 H}{\partial u^2} = 2(\eta _1 + \eta _2)> 0 \quad \text {and} \quad \frac{\partial ^2 H}{\partial v^2} = 2(\epsilon _1 + \epsilon _2) > 0. \end{aligned}$$
