# Supplementary material for: The effect of governance structures on optimal control of two-patch epidemic models
Source: J Math Biol. 2023 Oct 20;87(5):74. doi: 10.1007/s00285-023-02001-8 (PMC10589198; doi:10.1007/s00285-023-02001-8)

Appendix C. Supplementary tables

Model	Control type	Patch	Cost of vaccination	Cost of second control	Total cost
Cholera	Non-uniform	1	5,741.02	1,333.09	33,464.58
	Non-uniform	2	7,643.46	1,587.80	39,203.21
	Uniform	1	6,035.83	1,457.47	33,566.18
	Uniform	2	7,464.92	1,457.47	39,230.94
Ebola	Non-uniform	1	1,271.79	1,084.12	8,092.78
	Non-uniform	2	1,063.24	474.03	2,804.26
	Uniform	1	1,189.45	993.16	8,016.82
	Uniform	2	1,189.45	707.83	2,982.62

Table C.4: Cost totals from the simulations of the cholera and Ebola models under non-uniform and uniform policies after the start of controls (60 days for cholera and 150 days for Ebola). Because the cost coefficients for infections are set to one for each model, the cost of infections are equal to the total number of infections given in Table 2. The costs of vaccination, hospitalization and sanitation listed here do not include non-linear costs associated with control. The second control for cholera gives the (scaled) volume of contaminated water that was sanitized over the simulation period. The second control for Ebola gives the total number of hospitalizations over the simulation period. As we assume no common units between the cholera and Ebola models, the cost totals between the two models are not comparable.

Appendix D. Supplementary figures

Figure D.9: Trajectories of the cholera model epidemiological compartments without control.

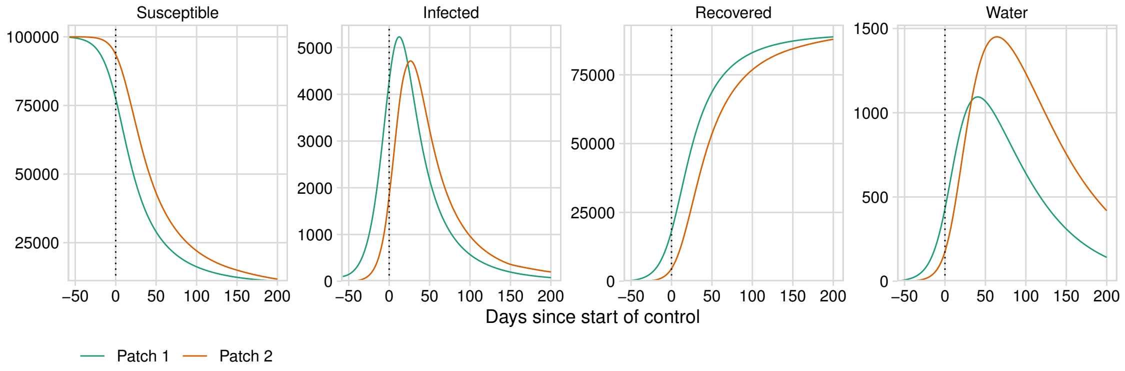

Figure D.10: Trajectories of the Ebola model epidemiological compartments without control.

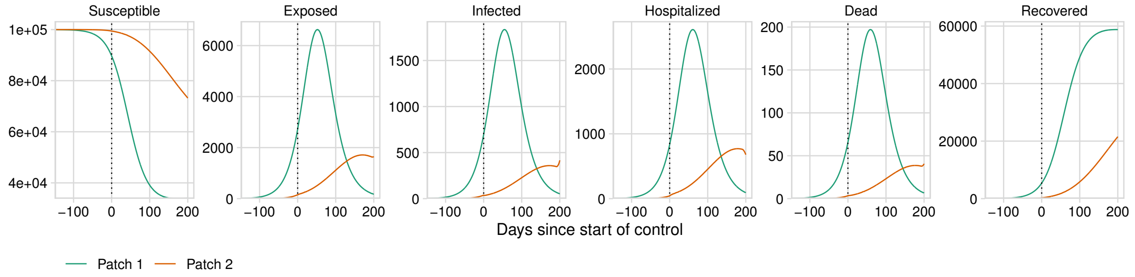

Figure D.11: The effect of increasing cost parameters on the optimal control trajectories of the Ebola model. Cost parameters are increased by one order of magnitude from baseline: (a) increasing linear cost of vaccination in Patch 1 from $A_1 = 0.01$ to 0.1; (b) increasing linear cost of hospitalization in Patch 1 from $B_1 = 0.1$ to 1; (c) increasing linear cost of hospitalization in Patch 2 from $B_2 = 0.1$ to 1. The case where the cost of vaccination in Patch 2 is increased is not shown as this simulation did not converge for the chosen the control response time of 150 days. Total cost of vaccination and hospitalization under each scenario is (a) non-uniform: 5870 and uniform: 6,009, (b) non-uniform: 3,976 and uniform: 4,264, and (c) non-uniform: 7,795 and uniform: 8,066 (compared to the baseline case, with cost of non-uniform: 3,893 and uniform: 4,080).

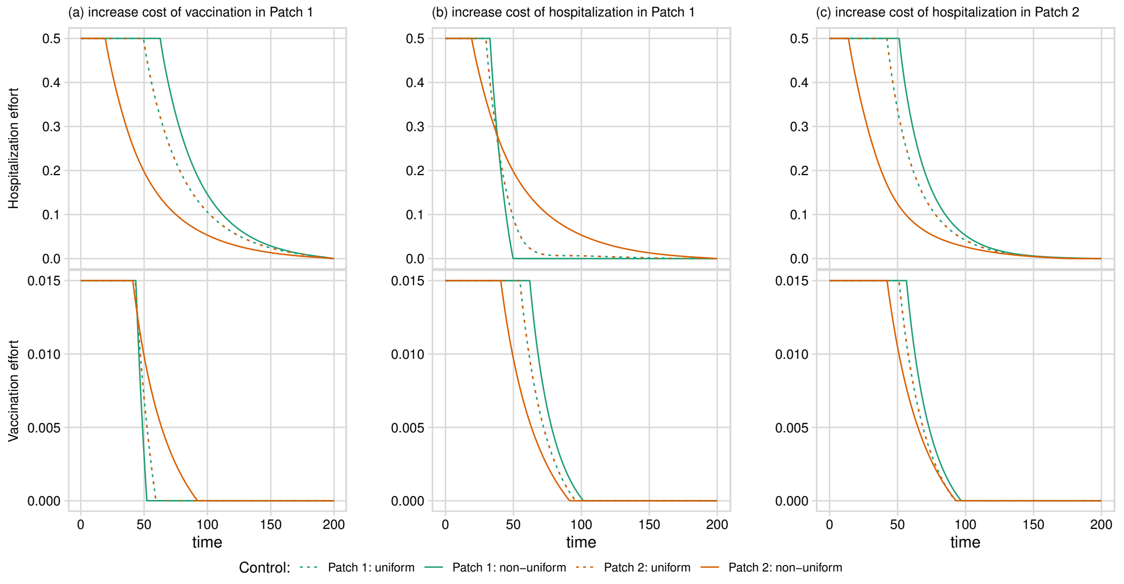

Figure D.12: The effect of increasing movement parameters on the optimal control trajectories of the Cholera model. Movement parameters are increased by one order of magnitude: (a) baseline case shown in main text, where movement rate $m_1 = 5 \times 10^{-4}$ and $m_2 = 5 \times 10^{-4}$; (b) increasing movement rate from Patch 1 to Patch 2, $m_1 = 5 \times 10^{-3}$; (c) increasing movement rate from Patch 2 to Patch 1 $m_2 = 5 \times 10^{-3}$; (d) increasing movement in both directions, $m_1 = 5 \times 10^{-3}$ and $m_2 = 5 \times 10^{-3}$.

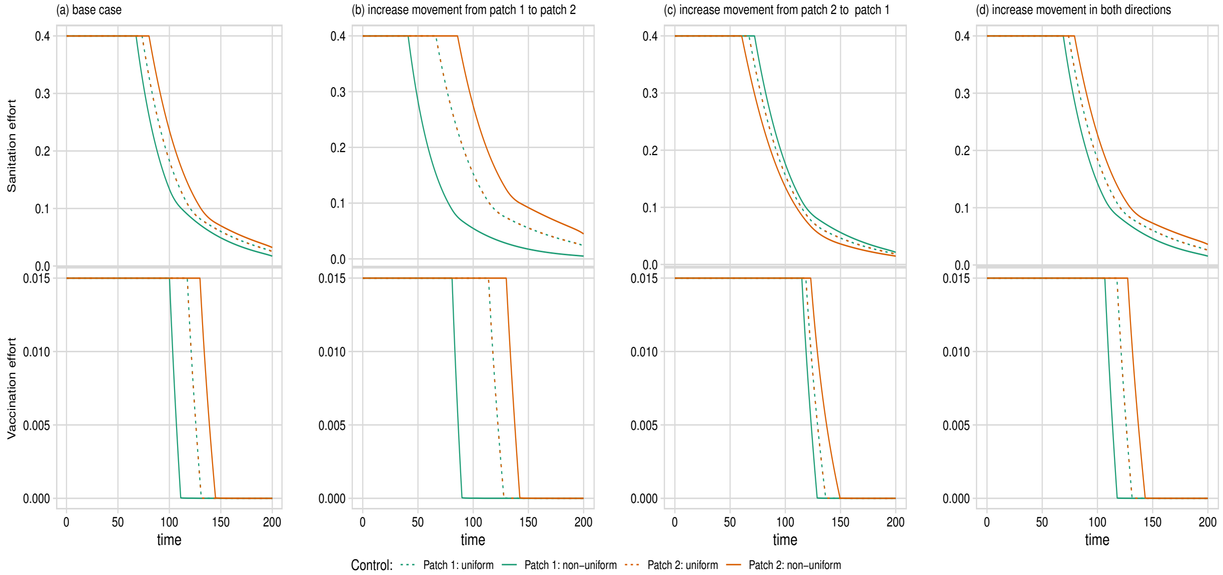

Supplement: Supplementary file 1 — (pdf 1082 KB) [file 285_2023_2001_MOESM1_ESM.pdf]
